# Bibliometric analysis and initial animal efficacy evaluation of top ten scoring drugs to enhance oral rehydration therapy in early post-burn shock

**DOI:** 10.3389/fphar.2025.1614159

**Published:** 2025-08-18

**Authors:** Xiang-Yu Liu, Yu-Shou Wu, Yi-Rui Qu, Hui Zhou, Tian Liu, Xiao-Wei Su, Fang-Chao Hu, Jin-Guang Zheng, Shao-Fang Han, Jia-Ke Chai, Yun-Fei Chi

**Affiliations:** 1 Senior Department of Burns & Plastic Surgery, Institute of Burn in the Fourth Medical Centre, Chinese PLA General Hospital, Beijing, China; 2 Graduate School, Chinese PLA General Hospital, Beijing, China

**Keywords:** burn, shock, oral rehydration therapy, world health organization-recommended oral rehydration solution, drug screening, bibliometric analysis, animal experiment, clinical application

## Abstract

**Background/objectives:**

Burns can cause severe physiological disturbances. Oral rehydration therapy (ORT) is an alternative to intravenous fluids. However, the World Health Organization-recommended oral rehydration solution (WHO-ORS) lacks specific components to address the critical physiological changes in patients with burns. This study aimed to identify and evaluate several drugs that enhance the ORT efficacy in burn shock management.

**Methods:**

A systematic search of PubMed, Web of Science, and Scopus (2000.01.01–2024.06.30) yielded 1,500 relevant studies, from which 270 were selected for bibliometric analysis. Drug candidates (≥3 mentions) were prioritized via the Bibliometric Evidence Score (BES) integrating publication frequency, journal impact factor (5-year average), impact score, and Q1 journal distribution. Subsequently, the translational potential of these candidates was assessed using an Integrated Translational Score incorporating weighted dimensions: Mechanistic Clinical Alignment Score (weight = 0.45), Emergency Deployment Feasibility (weight = 0.20), and BES (weight = 0.35). The top 10 drugs by the BES were selected for experimental validation, which were tested in a rat model with 50% total body surface area full-thickness burns (n = 286, 22/group), comparing sham controls, untreated controls, WHO-ORS, and drug-adjuvanted ORS groups. Primary outcomes included 48 h survival rate and blood lactate (Lac), hematocrit (HCT), malondialdehyde (MDA), and interleukin-6 (IL-6) levels.

**Results:**

Teprenone or vitamin C in combination with the WHO-ORS significantly improved survival outcomes following severe burns. They reduced blood lactate, HCT, MDA, and IL-6 levels. Glutamine and ethyl pyruvate showed beneficial effects but did not significantly improve survival. Hypertonic Saline and Dobutamine failed to demonstrate efficacy.

**Conclusion:**

This study demonstrated that adding teprenone or vitamin C to the WHO-recommended ORS can enhance the therapeutic efficacy of ORT in managing burn shock. These findings provide a scientific basis for further clinical trials and development of optimized ORS for patients with burns.

## 1 Introduction

Burn injuries pose significant threats to patient health and survival, leading to a range of physiological disturbances, including tissue damage, immune suppression, and metabolic disorders (Żwierełło et al., 2023). Among these complications, burn shock is particularly severe, often resulting in hypovolemia, ischemia, hypoxia, and oxidative stress, which can profoundly affect patient outcomes and increase mortality and morbidity rates (Cartotto et al., 2022). Traditional intravenous (IV) fluid resuscitation, which is highly effective in emergencies, has several limitations. It requires medical equipment, trained personnel, and a sterile environment, which may not be readily available in resource-limited settings (Cancio et al., 2017). Additionally, IV therapy carries risks, such as infection and thrombosis (Ogston-Tuck, 2012). In contrast, oral rehydration therapy (ORT) offers a cost-effective, convenient, and widely accessible alternative. ORT can be administered without the need for specialized equipment and has been successfully used for various medical conditions, including dehydration due to diarrhea (Glass and Stoll, 2018). However, the current World Health Organization-recommended oral rehydration solution (WHO-ORS) lacks specific components to address the multifaceted physiological disturbances associated with burn shock (Liu X. Y. et al., 2024). Burn shock is characterized by significant hypovolemia due to fluid loss and increased capillary permeability; widespread ischemia and hypoxia, particularly in the gastrointestinal tract; and oxidative stress due to the production of reactive oxygen species (ROS) during the reperfusion phase (Jeschke et al., 2020). Intestinal mucosal injury and increased permeability further contribute to systemic inflammation and the risk of sepsis (Di Vincenzo et al., 2024). Given these challenges, identifying and evaluating clinically approved drugs that could enhance the therapeutic efficacy of ORT in the management of early post-burn shock are critically needed.

This study aimed to address this need by conducting a systematic literature review and initial animal experiments to determine the most effective drug(s) for improving outcomes in patients with early post-burn shock. The findings of this study could provide a scientific basis for developing optimized ORS, offering a new and improved strategy for treating patients with burns. By addressing the limitations of the current ORS and providing a more comprehensive treatment approach, this study has the potential to significantly reduce the mortality and morbidity rates and improve the overall recovery of patients with burns, particularly in settings where IV therapy is not readily available.

## 2 Materials and methods

### 2.1 Literature search and screening

To identify potential drugs for enhancing ORT for preventing and treating burn shock and its associated pathophysiological mechanisms, a systematic literature search was conducted in PubMed, Web of Science, and Scopus databases. Studies published between January 1, 2000, and June 30, 2024, were included. All data were obtained from public databases and did not include human participants; thus, ethical consent was not required. To minimize bias introduced by frequent database updates, all searches were completed on a single day, June 30, 2024.

**FIGURE 1:**
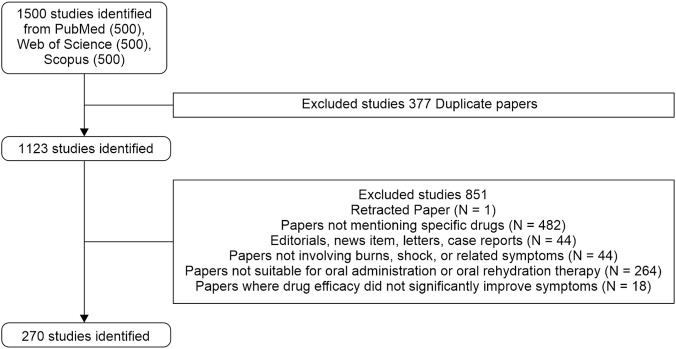
Flowchart of literature screening process.

The search strategy employed the following terms: TS = [burn OR shock OR (thermal injury) OR (hypovolemic shock)] AND TS = [ischemia OR hypoxia OR (oxidative stress) OR (reperfusion injury)] AND TS = [gastrointestinal OR (gut protection) OR (gut barrier) OR intestinal OR stomach OR (oral rehydration) OR (oral fluid therapy) OR (oral hydration) OR (oral resuscitation) OR (oral rehydration solution) OR (oral electrolyte solution) OR (oral rehydration therapy) OR (oral fluid replacement)]. We selected the top 500 most relevant articles from each database for a total of 1,500 articles. The following exclusion criteria were applied: (1) retracted papers; (2) studies not mentioning specific drugs; (3) editorials, news items, letters, and case reports; (4) studies unrelated to burns, shock, or related symptoms; (5) studies involving treatments unsuitable for oral administration or ORT; and (6) studies in which drug efficacy did not significantly improve symptoms. The detailed literature selection and screening process is illustrated in Figure 1.

### 2.2 Data extraction and management

Two reviewers (XYL and YSW) independently conducted the primary search and extracted data from all eligible studies. The extracted data included titles, journals, publication dates, countries and regions, authors, keywords, digital object identifier (DOI) numbers, latest impact factors of journals, average impact factors of journals over the past 5 years, journal citation report (JCR) quartile rankings, and total number of citations. Data were managed using Microsoft Excel 2016 (Redmond, Washington, United States). Data extraction and bibliometric mapping were conducted using VOSviewer software (v1.6.19, Leiden University, Leiden, Netherlands) and Stork software (https://www.storkapp.me). Statistical analyses of publication metrics were performed using GraphPad Prism 8 (GraphPad Software Inc., San Diego, CA, United States).

### 2.3 Bibliometric analysis

Bibliometric analysis was performed to evaluate the research trends and intellectual structures, encompassing performance analysis of publication patterns and science mapping of conceptual relationships. Performance analysis quantified contributions based on annual publication trends, geographic distribution of research output, journal impact factors (5-year average), and citation frequency. Science mapping employed VOSviewer software to visualize conceptual networks through keyword co-occurrence analysis (minimum 20 occurrences) and co-authorship relationships. Thematic clusters and collaboration networks were identified using VOSviewer’s clustering algorithms.

### 2.4 Drug screening and translational evaluation

#### 2.4.1 Evidence strength assessment

Drugs mentioned in at least three included studies were evaluated through a Bibliometric Evidence Score (BES), integrating four equally weighted parameters: (1) frequency of mention, (2) sum of impact scores for all papers referencing the drug, (3) number of publications in JCR Q1 journals, and (4) mean 5-year impact factor of journals publishing relevant studies. The Impact Score for each paper was defined as [LN (1 + Number of citations) + (Journal Impact Factor/Age of the paper)]. Raw values were normalized by Z-score transformation; BES was calculated by summing normalized values weighted equally (0.25 per parameter). The top 10 drugs by BES were selected for experimental validation.

#### 2.4.2 Comprehensive clinical-translational assessment

To evaluate clinical relevance and practical deployment potential, drugs cited in ≥3 studies were assessed through an integrated framework comprising three core dimensions: (1) BES: The foundational BES reflecting research robustness; (2) Mechanistic Clinical Alignment Score (MCAS): The MCAS for each drug was the sum of scores across all its supporting studies. The MCAS of each study quantifying clinical evidence through tiered evaluation (Tier I: 10 points per randomized clinical trials (RCT) including patients with burn/shock with oral administration; Tier II: 5 points per RCT for non-burn conditions involving ischemia, hypoxia, or reperfusion injury (core pathophysiology of burn shock); Tier III: 2 points per large animal study (swine, dog, or other non-rodent mammals); Tier IV: 1 point per rodent study; Tier V: 0.5 points per human primary cell or organoid study; Tier VI: 0 points for non-research literature; (2) Emergency Deployment Feasibility (EDF): Formulation viability scoring (Tier A: 3 points for approved oral formulations in regulatory databases including Food and Drug Administration in US/European Medicines Agency/National Medical Products Administration in China/Pharmaceuticals and Medical Devices Agency in Japan; Tier B: 2 points for compounds lacking approved formulations but amenable to simple compounding with moderate shelf life; Tier C: 1 point for compounds requiring complex compounding or exhibiting poor stability). These dimensions were synthesized into an Integrated Translational Score (ITS): ITS = [Z-score (MCAS) × 0.45] + [Z-score(EDF) × 0.2] + [Z-score (BES) × 0.35].

### 2.5 Animal experiments

To validate the efficacy of the top 10 drugs identified through bibliometric analysis and drug screening, animal experiments were conducted using a rat model of 50% total body surface area (TBSA) third-degree burn injury. A total of 286 male Wistar rats weighing 200–220 g were obtained from SPF Biotechnology Co., Ltd. (Beijing, China). The animals were housed under controlled conditions with a 12-h light/dark cycle and acclimatized for at least 1 week before the experiment. All experimental procedures were approved by the Animal Ethical Committee of the Fourth Medical Center of the PLA General Hospital (ethics approval ID: 2024KY058-KS001) and followed the guidelines of the National Institutes of Health for the care and use of laboratory animals. The rats were randomly divided into 13 groups (n = 22 per group): sham, burn (untreated control), WHO-ORS, and 10 drug-adjuvanted ORS groups.

Prior to anesthesia, the dorsal and ventral surfaces of the rats were shaved to ensure proper exposure to the burn injury. Anesthesia was induced via intraperitoneal injection of sodium pentobarbital (30 mg/kg). Except for the sham group, all rats underwent a 50% TBSA full-thickness burn. The dorsal and ventral surfaces were exposed to boiling water (94 °C) for 12 and 6 s, respectively. The Sham group was immersed in warm water (37 °C) for the same duration. After burn induction, the rats were dried gently, and the burn wounds were treated with 1% iodine tincture, which served both as a disinfectant and temporary analgesic. The animals were then returned to their cages, covered with large cotton pads, and provided with post-burn thermoregulation using a small animal heating pad to optimize recovery.

### 2.6 Oral liquid preparation and resuscitation method

The WHO-ORS solution was prepared by dissolving one packet of WHO-ORS powder (5.125 g, containing sodium chloride 0.65 g, potassium chloride 0.375 g, sodium citrate 0.725 g, and anhydrous glucose 3.375 g) in 250 mL of potable water. For the 10 drug-adjuvanted ORS solutions, each drug was added to 1,000 mL of potable water along with four packets of WHO-ORS powder. Drug concentrations were based on either standard clinical dosages or the most commonly used experimental doses, as determined from a literature review. Rehydration volumes were calculated based on the Parkland formula and halved to determine the total volume for this study. Rehydration began immediately post-injury, with gavage administration every 2 h over an 8-h period (a total of 5 doses), followed by *ad libitum* water access.

### 2.7 Outcome measures

In each group, 10 rats were designated for the observation of 48 h survival rates, while the remaining 12 rats were used for blood sample collection at 6 h and 24 h post-injury (n = 6 in each group at each time point). The primary outcomes measured included the 48 h survival rate and lactate (Lac), hematocrit (HCT), malondialdehyde (MDA), and interleukin-6 (IL-6) levels. Lac levels were measured using an i-STAT System 300 (Abbott Laboratories Inc., NY, United States), HCT levels were determined using a Mindray BC-3000 Plus automated hematology analyzer (Mindray Bio-medical Electronics Co., Ltd., Shenzhen, China); MDA and IL-6 levels were quantified using commercially available ELISA kits (MDA: Nanjing Jiancheng Bioengineering Institute, Nanjing, China; IL-6: Abcam, Cambridge, MA, United States).

### 2.8 Statistical analysis

Statistical analyses were performed using SPSS software (version 23.0; IBM Corp., Armonk, NY, United States). Survival analyses were conducted using the Kaplan-Meier method, followed by the log-rank test to compare survival curves among the groups. Continuous variables (blood lactate, HCT, MDA, and IL-6 levels) are expressed as mean ± standard deviation (SD), and comparisons between groups were performed using one-way ANOVA followed by Tukey’s *post hoc* test with equal variances, while Dunnett’s T3 test with unequal variances. For correlation analysis between bibliometric and translational metrics, Spearman’s rank correlation was employed to assess the concordance between the BES and ITS rankings across all drugs with occurrence frequency ≥3. Statistical significance was defined as *P* value <0.05.

## 3 Results

### 3.1 Integrated bibliometric analysis

**FIGURE 2:**
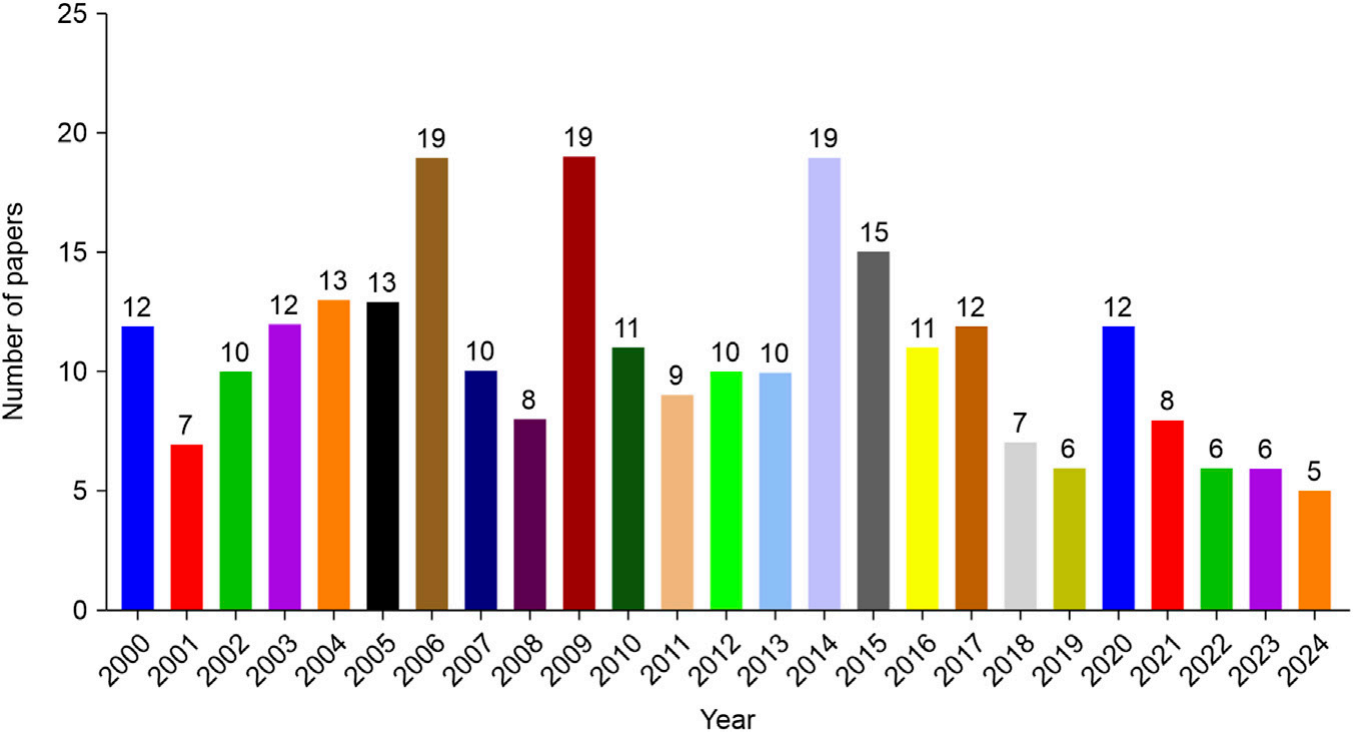
Annual publication trends of included studies.

**FIGURE 3:**
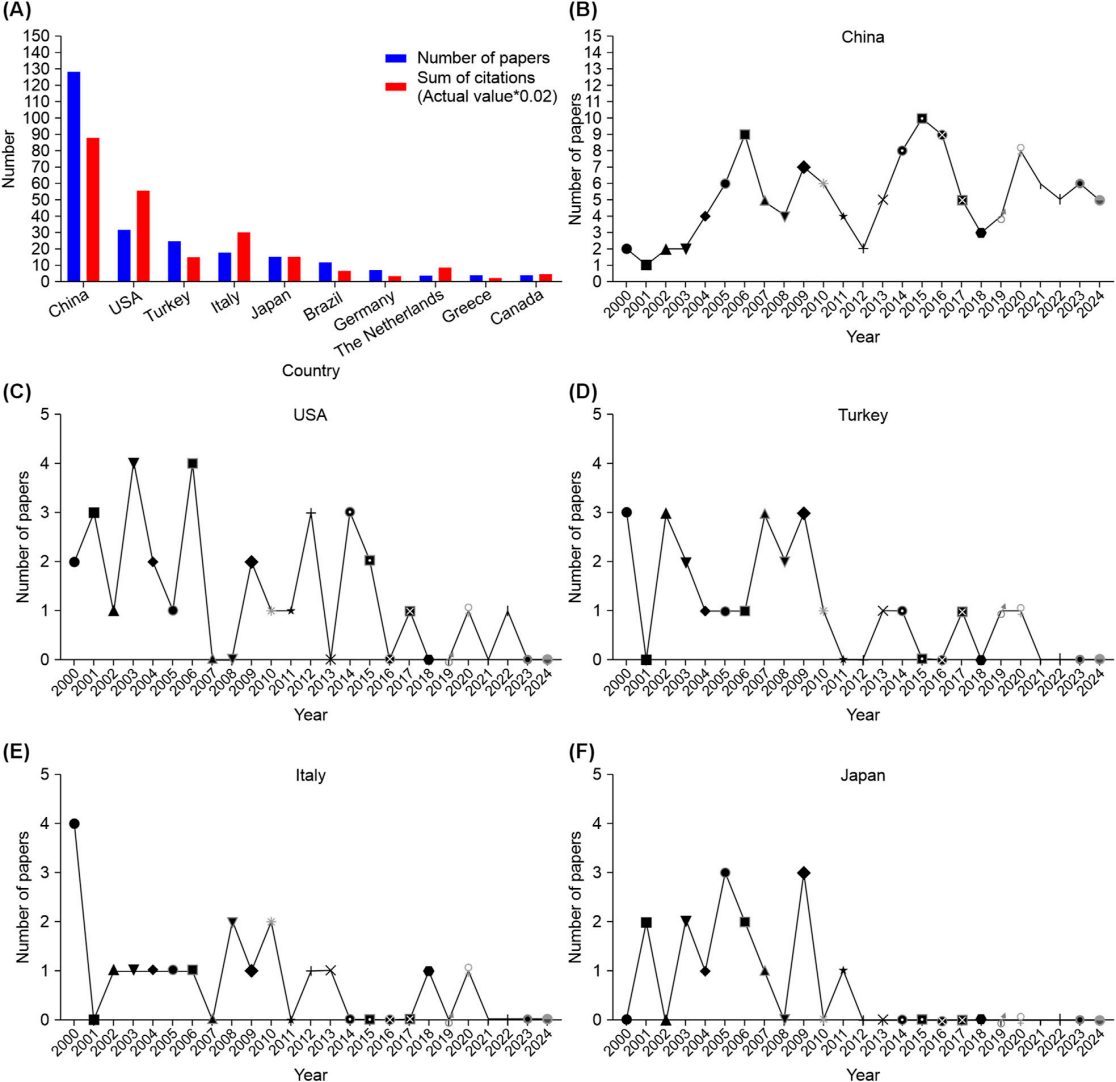
Global contributions and temporal trends in research on pathophysiological mechanisms and therapeutic strategies for burn, shock, and associated gastrointestinal interventions. **(A)** The number of publications, and citation frequency (×0.02) in the top 10 countries or regions. **(B)** Annual publication trends in China. **(C)** Annual publication trends in United States. **(D)** Annual publication trends in Turkey. **(E)** Annual publication trends in Italy. **(F)** Annual publication trends in Japan.

Systematic screening yielded 270 eligible studies (Figure 1). Annual publication analysis revealed fluctuating outputs from 2000 to 2024 (Figure 2), peaking in 2006, 2009, and 2014 (19 publications each). Geospatial distribution demonstrated China’s dominance with 128 publications (47.4%), followed by the United States (32 publications, 11.9%) and Italy (18 publications, 6.7%) (Figure 3A). Notably, Italy exhibited exceptional citation impact (1,504 citations; 83.6 citations/paper) despite moderate output volume, indicating disproportionate scientific influence. Longitudinal national trends (Figures 3B–F) revealed China’s sustained growth post-2010 versus Japan’s research discontinuation after 2012.

**FIGURE 4:**
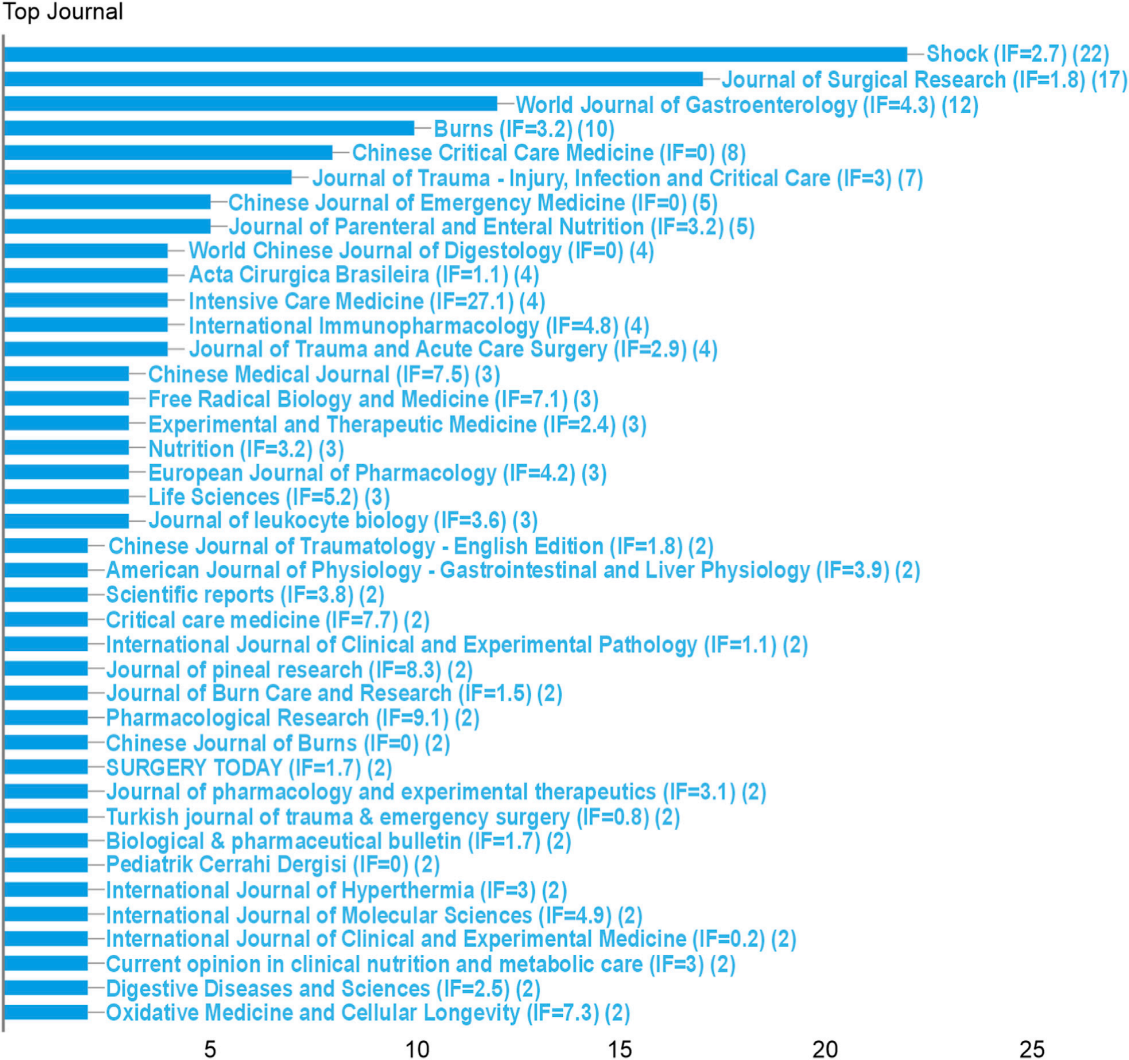
Top 40 journals by publication volume.

**FIGURE 5:**
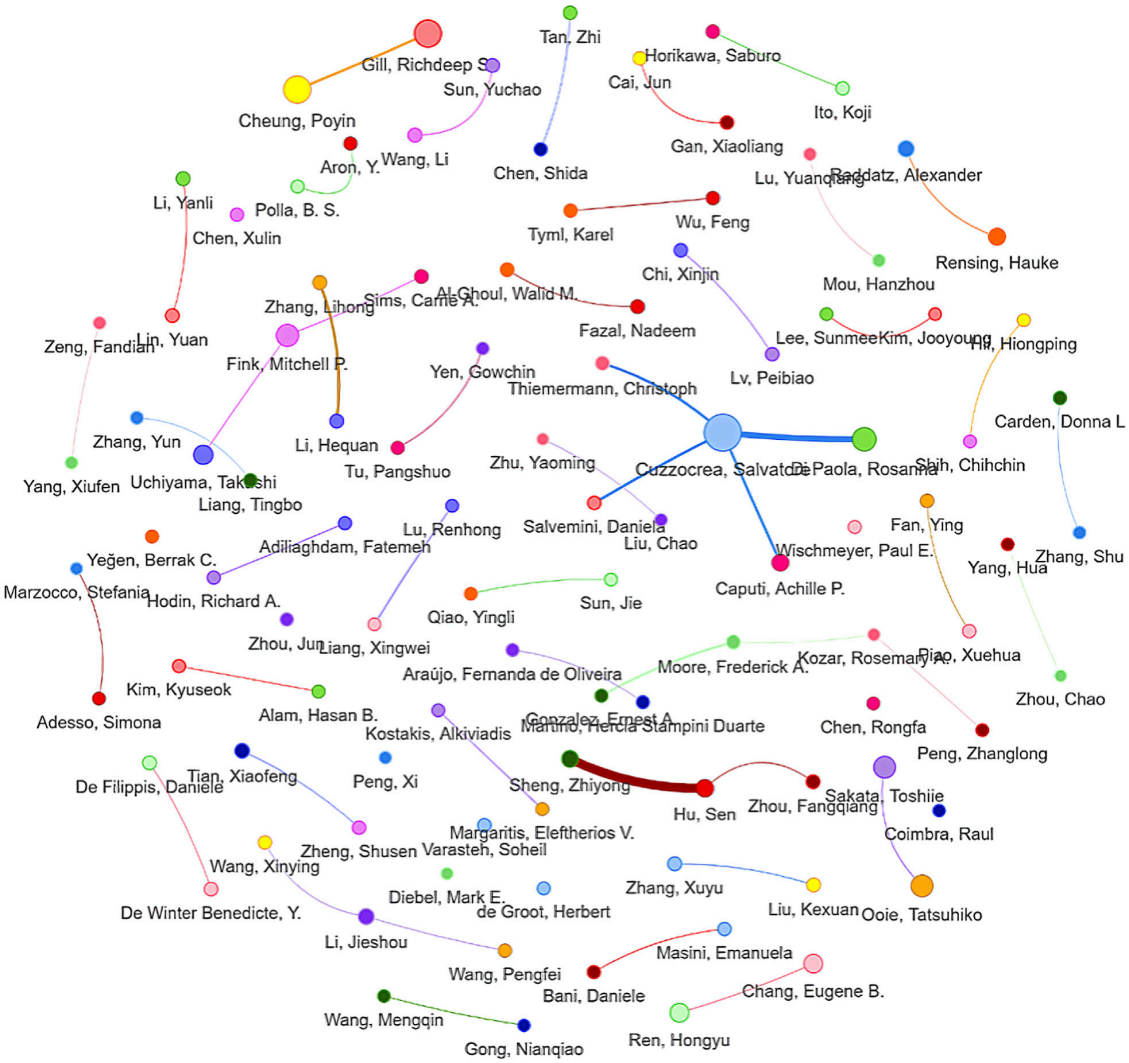
Co-authorship network of researchers.

Journal analysis identified *Shock* (IF = 2.9; n = 22), *Journal of Surgical Research* (IF = 1.7; n = 17), and *World Journal of Gastroenterology* (IF = 5.4; n = 12) as primary knowledge dissemination venues (Figure 4). Author network mapping revealed three major collaborative clusters (Figure 5; Table 1), with Sheng Zhiyong (n = 16 publications; impact score = 19.0), Cuzzocrea Salvatore (n = 14; 87.6), and Hu Sen (n = 12; 20.9) as pivotal nodes.

**TABLE 1:** Top 10 authors with most publications in research scope of pharmacological interventions for burn, shock, or related symptoms, particularly those with potential applications in ORT.

Author	No. of publications	Total impact score	Country	Affiliation
Sheng Zhiyong	16	19	China	The First Hospital Affiliated to the PLA General Hospital
Cuzzocrea Salvatore	14	87.6	Italy	University of Messina
Hu Sen	12	20.9	China	The First Hospital Affiliated to the PLA General Hospital
Di Paola Rosanna	5	41.8	Italy	University of Messina
Li Jieshou	4	16.6	China	Medical School of Nanjing University
Moore Frederick A	4	12	United States	University of Florida
Fink Mitchell P	3	36.6	United States	University of Pittsburgh Medical School
Tian Xiaofeng	3	12.2	China	The Second Affiliated Hospital of Dalian Medical University
Thiemermann Christoph	3	12.2	United Kingdom	Queen Mary University of London
Zhou Fangqiang	3	9.5	United States	Fresenius Medical Care

**FIGURE 6:**
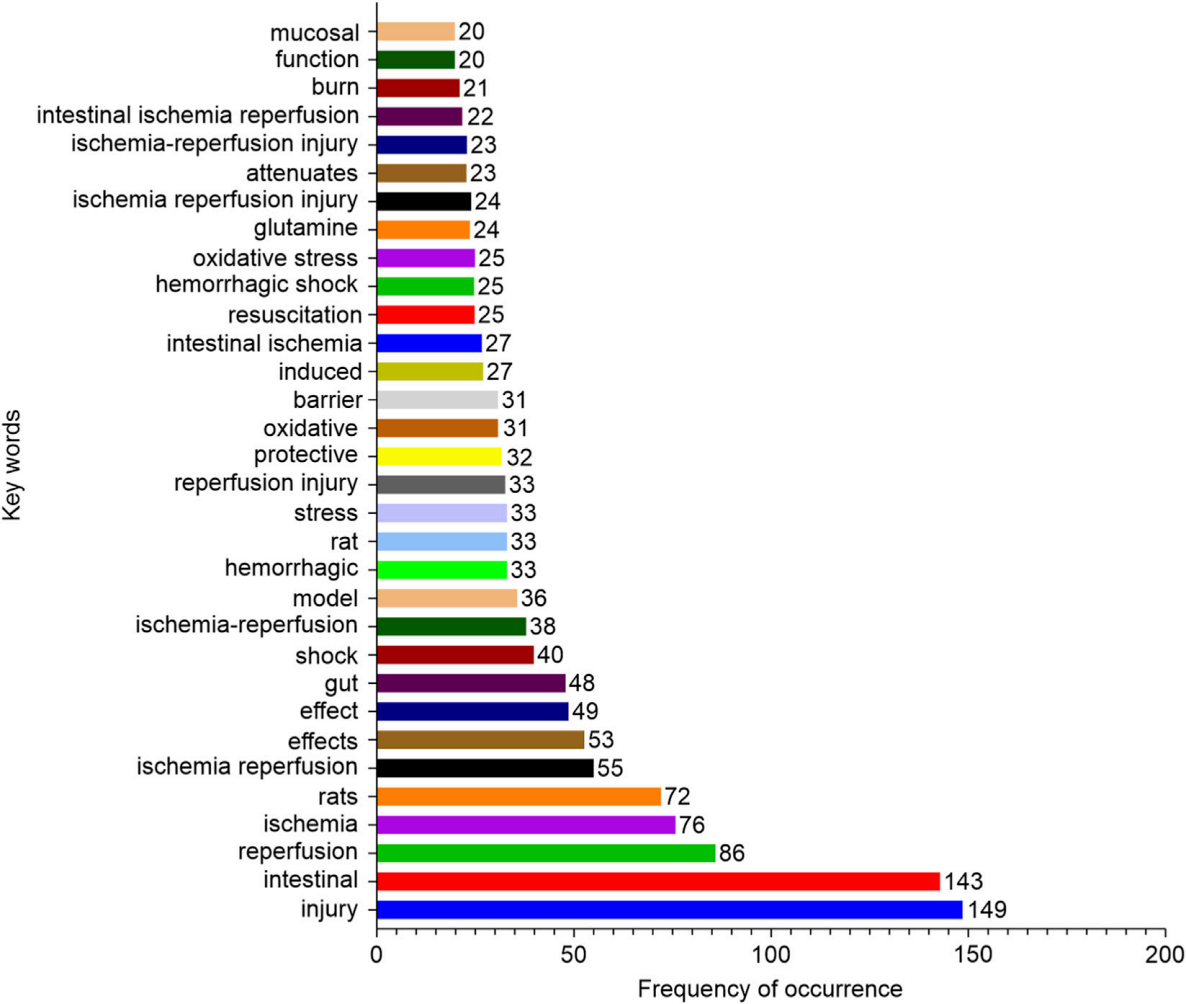
Frequency of keywords in included studies (frequency of occurrence ≥20).

**FIGURE 7:**
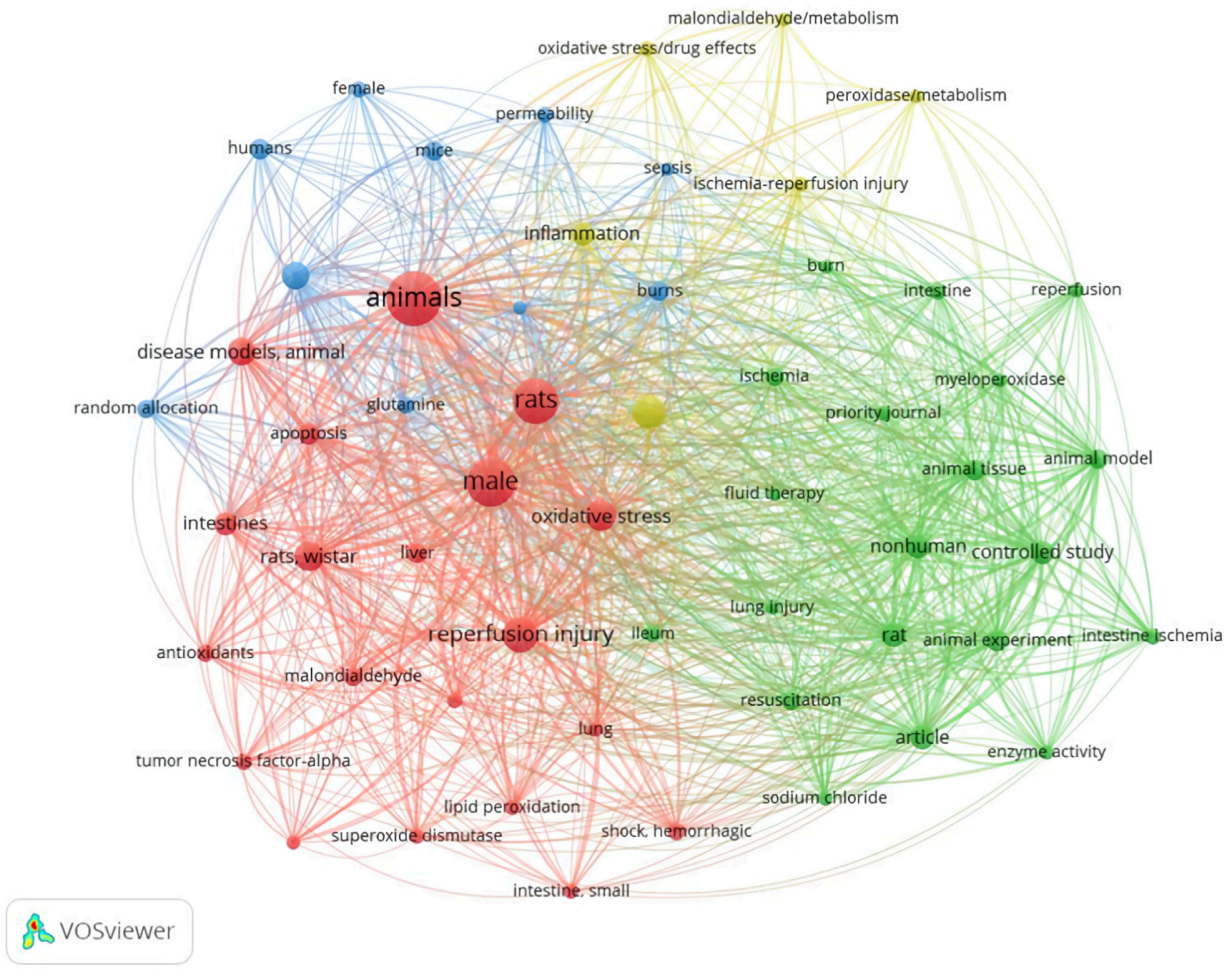
Co-occurrence network of keywords.

Keyword analysis demonstrated high-frequency terms including “injury” (149 occurrences), “intestinal” (143), and “reperfusion” (86). Cluster visualization revealed four interconnected domains: ischemia-reperfusion injury terms co-occurred with oxidative stress markers; fluid resuscitation concepts aligned with experimental models; intestinal barrier keywords clustered with bacterial translocation terms; and inflammatory pathways linked to metabolic mediators (Figures 6, 7).

### 3.2 Drug screening and translational validation

#### 3.2.1 Bibliometric evidence score (BES) evaluation

**FIGURE 8:**
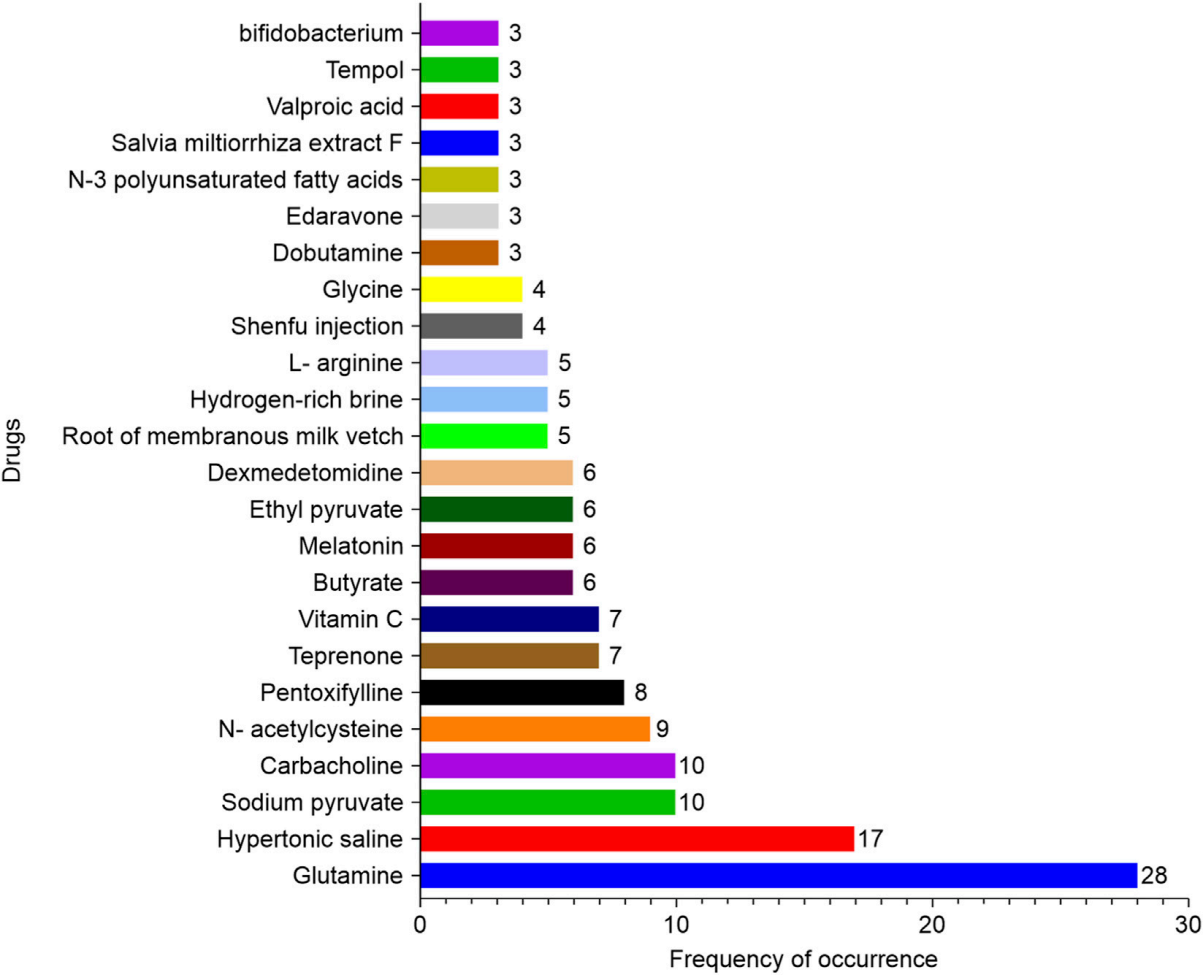
Drugs with a frequency of occurrence ≥3.

The BES was derived from the weighted integration of four standardized metrics, including the frequency of occurrence, impact scores, publication quality, and journal impact factors. Table 2 presents the detailed results of this evaluation, listing all drugs with a frequency of occurrence ≥3 (Figure 8, n = 24) in descending order of their BES. Among the evaluated drugs, glutamine emerged as the top candidate, with a BES of 2.546, supported by its high frequency of occurrence (28), substantial sum of impact scores (108.176), and significant number of JCR Q1 publications (10). Subsequent high-ranking candidates included hypertonic saline (BES = 1.046), butyrate (BES = 0.711), melatonin (BES = 0.629), teprenone (BES = 0.586), sodium pyruvate (BES = 0.282), N-acetylcysteine (BES = 0.214), ethyl pyruvate (BES = 0.192), vitamin C (BES = 0.146), and dobutamine (BES = 0.070). These 10 drugs were advanced to experimental validation based on their comprehensive bibliometric profiles.

**TABLE 2:** Bibliometric evidence score of drugs (frequency of occurrence ≥3).

Drugs	Frequency of occurrence	Z-Score of frequency of occurrence	Sum of impact scores	Z-Score of sum of impact scores	No. of JCR Q1 publications	Z-Score of no. of JCR Q1 publications	Average 5-year impact factors	Z-Score of average 5-year impact factors	Bibliometric evidence score
Glutamine	28	3.811	108.176	4.020	10	2.904	2.743	−0.553	2.546
Hypertonic saline	17	1.831	56.087	1.520	7	1.629	2.247	−0.795	1.046
Butyrate	6	−0.150	26.059	0.079	6	1.204	7.383	1.710	0.711
Melatonin	6	−0.150	27.007	0.125	6	1.204	6.617	1.336	0.629
Teprenone	7	0.030	27.009	0.125	4	0.354	7.643	1.837	0.586
Sodium pyruvate	10	0.570	29.863	0.262	5	0.779	2.890	−0.482	0.282
N- acetylcysteine	9	0.390	33.151	0.419	4	0.354	3.244	−0.309	0.214
Ethyl pyruvate	6	−0.150	26.496	0.100	2	−0.496	6.567	1.312	0.192
Vitamin C	7	0.030	26.438	0.097	4	0.354	4.086	0.102	0.146
Dobutamine	3	−0.690	9.354	−0.723	2	−0.496	8.367	2.190	0.070
Root of membranous milk vetch	5	−0.330	20.294	−0.198	4	0.354	4.120	0.118	−0.014
Dexmedetomidine	6	−0.150	22.084	−0.112	3	−0.071	4.117	0.117	−0.054
Pentoxifylline	8	0.210	27.198	0.134	3	−0.071	2.200	−0.818	−0.136
Edaravone	3	−0.690	12.067	−0.592	2	−0.496	5.200	0.645	−0.283
N-3 polyunsaturated fatty acids	3	−0.690	10.143	−0.685	2	−0.496	4.167	0.141	−0.432
Shenfu injection	4	−0.510	8.806	−0.749	3	−0.071	2.925	−0.464	−0.449
Carbacholine	10	0.570	22.211	−0.106	1	−0.921	0.680	−1.560	−0.504
Salvia miltiorrhiza extract F	3	−0.690	7.079	−0.832	2	−0.496	3.400	−0.233	−0.563
Valproic acid	3	−0.690	10.827	−0.652	2	−0.496	2.900	−0.477	−0.579
Hydrogen-rich brine	5	−0.330	19.611	−0.230	1	−0.921	2.000	−0.916	−0.599
Tempol	3	−0.690	10.340	−0.675	2	−0.496	2.567	−0.639	−0.625
Glycine	4	−0.510	18.026	−0.306	0	−1.346	2.750	−0.550	−0.678
L- arginine	5	−0.330	13.265	−0.535	1	−0.921	1.840	−0.994	−0.695
Bifidobacterium	3	−0.690	14.270	−0.487	0	−1.346	2.400	−0.721	−0.811

The drugs are ranked in descending order based on their comprehensive evaluation scores. The Z-score was used to standardize the raw data into a standard normal distribution with a mean of 0 and SD of 1. The formula used is as follows:

Z-score = (Raw value−Mean)/(Standard deviation).

The Impact Score for each publication was calculated using the following formula.

Impact Score = [LN (1 + Number of Citations) + (Journal Impact Factor/Age of the paper)].

#### 3.2.2 Translational validation analysis

To evaluate clinical relevance and deployment feasibility—critical considerations for practical implementation—24 candidate drugs underwent comprehensive translational assessment as detailed in Table 3. The MCAS ranged between 3 and 33 (median = 6.5), with glutamine achieving the highest score supported by Tier I RCT evidence in patients with burn. EDF analysis showed 66.7% of candidates (16/24) had moderate-to-high deployability (EDF ≥2). Significant concordance existed between BES and ITS rankings (Spearman’s ρ = 0.815, *P* < 0.001), with 80% of BES-top 10 drugs (8/10) demonstrating positive translational potential (ITS > 0).

**TABLE 3:** Comprehensive translational assessment of all candidate drugs (frequency of occurrence ≥3).

Drugs	Bibliometric evidence score	Bibliometric evidence score (BES) ranking	Mechanistic clinical alignment score	Z-Score of mechanistic clinical alignment score	Emergency deployment feasibility	Z-Score of Emergency deployment feasibility	Integrated translational score (ITS)	Integrated translational score ranking
Glutamine	2.546	1	33	3.819	3	0.768	2.763	1
Hypertonic saline	1.046	2	20	1.903	1	−1.536	0.915	2
Butyrate	0.711	3	5.5	−0.233	3	0.768	0.297	5
Melatonin	0.629	4	6	−0.160	3	0.768	0.302	4
Teprenone	0.586	5	3	−0.602	3	0.768	0.088	9
Sodium pyruvate	0.282	6	9	0.282	2	−0.384	0.149	8
N- acetylcysteine	0.214	7	9	0.282	3	0.768	0.356	3
Ethyl pyruvate	0.192	8	6	−0.160	1	−1.536	−0.312	17
Vitamin C	0.146	9	6.5	−0.086	3	0.768	0.166	7
Dobutamine	0.070	10	4	−0.454	1	−1.536	−0.487	20
Root of membranous milk vetch	−0.014	11	4.5	−0.381	3	0.768	−0.023	11
Dexmedetomidine	−0.054	12	5	−0.307	3	0.768	−0.003	10
Pentoxifylline	−0.136	13	8	0.135	3	0.768	0.167	6
Edaravone	−0.283	14	2.5	−0.675	3	0.768	−0.249	14
N-3 polyunsaturated fatty acids	−0.432	15	3	−0.602	3	0.768	−0.269	15
Shenfu injection	−0.449	16	6	−0.160	2	−0.384	−0.306	16
Carbacholine	−0.504	17	13.5	0.945	1	−1.536	−0.058	12
Salvia miltiorrhiza extract F	−0.563	18	3	−0.602	3	0.768	−0.314	18
Valproic acid	−0.579	19	3	−0.602	3	0.768	−0.320	19
Hydrogen-rich brine	−0.599	20	5	−0.307	1	−1.536	−0.655	23
Tempol	−0.625	21	3	−0.602	1	−1.536	−0.797	24
Glycine	−0.678	22	3	−0.602	2	−0.384	−0.585	21
L- arginine	−0.695	23	5.5	−0.233	3	0.768	−0.195	13
Bifidobacterium	−0.811	24	3	−0.602	2	−0.384	−0.631	22

The drugs are ranked in descending order based on their Bibliometric evidence score. The Z-score was used to standardize the raw data into a standard normal distribution with a mean of 0 and standard deviation of 1. The formula used is as follows:

Z-score = (Raw value−Mean)/(Standard deviation).

Spearman’s ρ = 0.82 (*P* < 0.001) for BES vs. ITS ranks.

### 3.3 Determination of experimental dosages for each drug

The experimental dose of each drug was determined based on previous studies conducted using rat models. For glutamine (Marzulene-S; Ajinomoto Co., Inc., Shanghai, China), a dosage of 1.0 g/kg/d was selected based on its demonstrated efficacy in stimulation of intestinal immunity and protection of intestinal integrity (Demirkan et al., 2008; Fan et al., 2009; Demirkan et al., 2010). Similarly, hypertonic saline was administered at a concentration of 7.5%, as recommended by other researchers, to significantly improve blood flow distribution (Kien et al., 1996). A butyrate dose (Aladdin, Shanghai, China) of 400 mg/kg/d was derived from a study by Zhou et al. (2022), which highlighted its role in the amelioration of burn-induced inflammation and intestinal injury. Regarding melatonin (Sigma, St. Louis, MO, United States), a dosage of 10 mg/kg was chosen based on its proven antioxidant properties (Sener et al., 2002; Tunali et al., 2005; Hristova et al., 2018). The remaining drugs, including teprenone (Eisai Co., Ltd., Tokyo, Japan, dosage: 200 mg/kg; Ooie et al., 2001; Zhu et al., 2005; Cao et al., 2015), sodium pyruvate (Sigma–Aldrich, St. Louis, MO, United States of America, dosage: 3.5 g per 1,000 mL ORS; Hu et al., 2014; Yu et al., 2015; Hu et al., 2016), N-acetylcysteine (Conba BioPharm.Co., Ltd., Zhejiang, China, dosage: 600 mg/kg/day; Saputro et al., 2018), ethyl pyruvate (Sigma-Aldrich, St. Louis, MO, United States, dosage: 40 mg/kg; Karabeyoğlu et al., 2008; Wang et al., 2016), vitamin C (Sigma-Aldrich, St. Louis, MO, United States, dosage: 66 mg/kg; Tanaka et al., 1999; Bark and Grände, 2014), and dobutamine (Sigma, St. Louis, MO, United States, dosage: 0.2 mg/kg; Hu and Sheng, 2002), were also determined based on established protocols from relevant literature.

### 3.4 Survival rate

**FIGURE 9:**
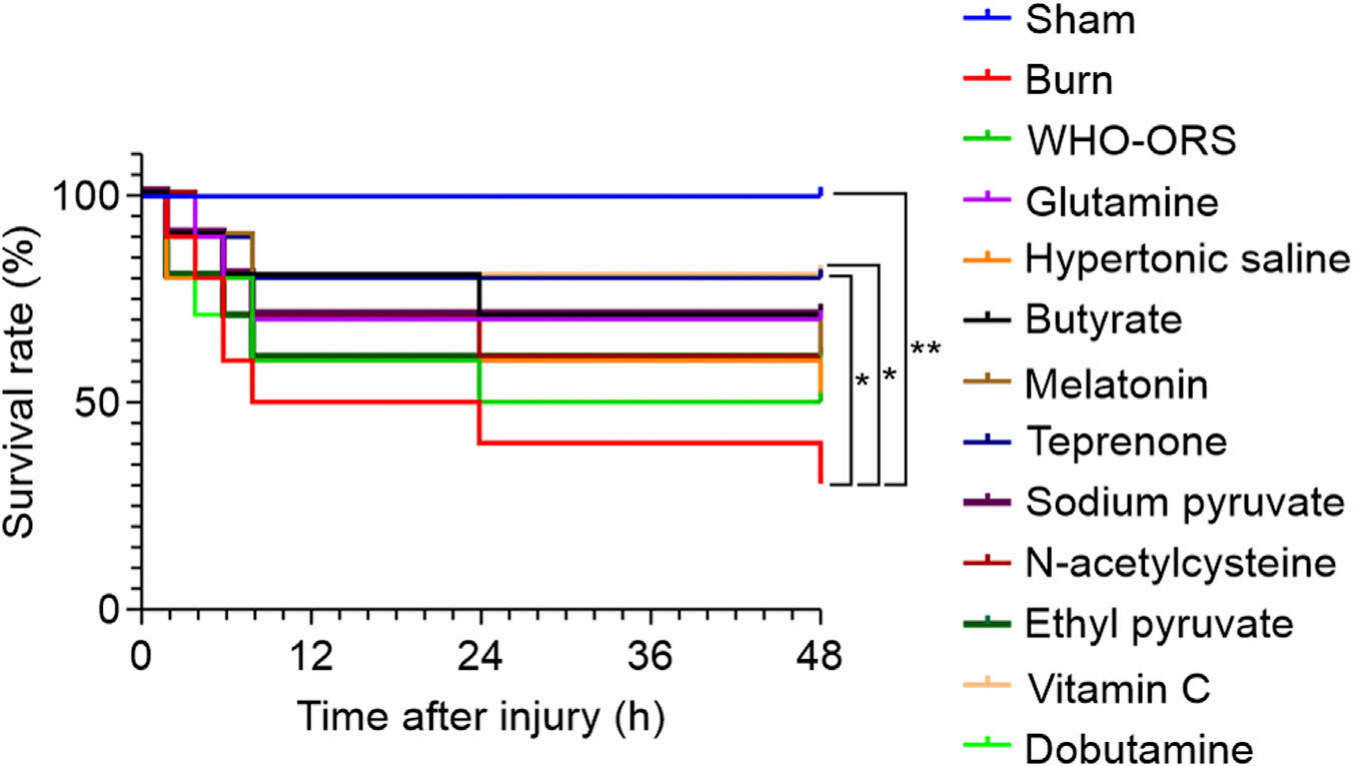
Survival analysis of rats in each group for 48 h (n = 10), compared with the Burn group: *P < 0.05, **P < 0.01.

The survival rate at 48 h post-injury was analyzed across all experimental groups. Figure 9 illustrates the Kaplan–Meier survival curves for all experimental groups. The survival rate was 100% in the sham group. In contrast, the burn group exhibited a significantly lower survival rate (30%), reflecting severe injury-induced mortality (*P* < 0.01). The WHO-ORS group demonstrated a moderate improvement in the survival rate (50%), which was higher than that in the burn group; however, the difference was not statistically significant (*P* > 0.05). Among the 10 therapeutic agents evaluated, glutamine (70%), butyrate (70%), melatonin (60%), teprenone (80%), sodium pyruvate (70%), N-acetylcysteine (60%), ethyl pyruvate (60%), and vitamin C (80%) showed higher survival rates compared to both the burn and WHO-ORS groups. However, hypertonic saline (50%) and dobutamine (50%) did not demonstrate any improvement compared to the WHO-ORS group.

Log-rank analysis revealed that only the teprenone and vitamin C groups achieved statistically significant improvements in survival rates compared with the burn group (*P* < 0.05). No other treatment groups showed statistically significant differences in survival rates compared with either the burn or WHO-ORS group (*P* > 0.05).

### 3.5 Biochemical indicators: Lac, HCT, MDA and IL-6

**FIGURE 10:**
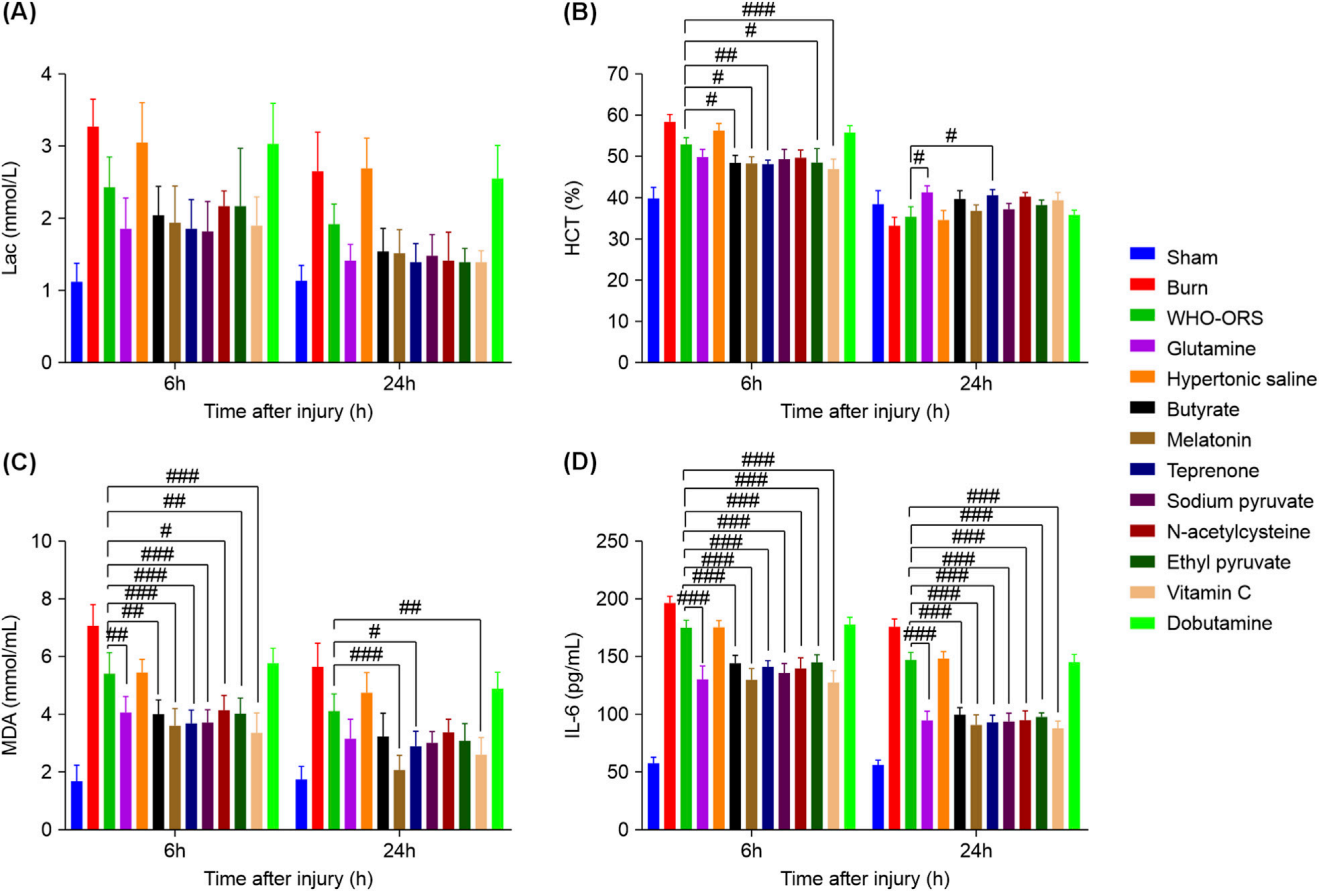
Changes in biochemical indicators at different time points in different groups. **(A)** Lac levels in mmol/L. **(B)** HCT levels in %. **(C)** MDA levels in nmol/mL. **(D)** IL-33 levels in pg/mL. Values are expressed as mean ± SD (n = 10). Statistical analysis was performed using one-way ANOVA followed by Tukey’s HSD test or Dunnett’s T3 *post hoc* test. Compared with WHO-ORS group: ^#^ *P* < 0.05, ^##^ *P* < 0.01, ^###^ *P* < 0.001.

Biochemical indicators, including Lac, HCT, MDA, and IL-6, were measured to assess the physiological and inflammatory responses and tissue perfusion post-injury. The results are summarized in Table 4 and Figure 10; the detailed analyses of each indicator are presented below.

**TABLE 4:** Blood lactate (Lac, mmol/L), hematocrit (HCT, %), malondialdehyde (MDA, nmol/mL), and interleukin-6 (IL-6, pg/mL) levels at different time points in different groups (mean ± standard deviation, n = 6).

Groups	Time Points	Lac (mmol/L)	HCT (%)	MDA (nmol/mL)	IL-6 (pg/mL)
Sham	6 h	1.118 ± 0.252***###	39.95 ± 2.669***###	1.697 ± 0.582***###	57.994 ± 4.32***###
	24 h	1.138 ± 0.196*#	38.55 ± 3.182	1.758 ± 0.44***###	56.142 ± 4.543***###
Burn	6 h	3.277 ± 0.383	58.433 ± 1.788##	7.091 ± 0.718###	196.923 ± 6.024##
	24 h	2.653 ± 0.534	33.317 ± 1.899	5.667 ± 0.833##	175.933 ± 6.699###
WHO-ORS	6 h	2.44 ± 0.408	53.083 ± 1.533**	5.455 ± 0.699***	175.761 ± 5.947**
	24 h	1.922 ± 0.279	35.65 ± 2.199	4.152 ± 0.58**	147.509 ± 5.952***
Glutamine	6 h	1.845 ± 0.435***	50.033 ± 1.775***	4.091 ± 0.548***##	130.354 ± 11.645***###
	24 h	1.413 ± 0.226*	41.483 ± 1.416**#	3.151 ± 0.687***	93.92 ± 8.887***###
Hypertonic saline	6 h	3.063 ± 0.551	56.35 ± 1.718	5.485 ± 0.437***	175.76 ± 6.037**
	24 h	2.702 ± 0.409	34.8 ± 2.237	4.788 ± 0.677	148.925 ± 5.751***
Butyrate	6 h	2.045 ± 0.396**	48.683 ± 1.649***#	4.03 ± 0.48***##	144.649 ± 6.538***###
	24 h	1.545 ± 0.309	39.783 ± 2.037*	3.273 ± 0.754***	99.003 ± 6.33***###
Melatonin	6 h	1.935 ± 0.51***	48.483 ± 1.634***#	3.637 ± 0.586***###	130.23 ± 9.201***###
	24 h	1.513 ± 0.323	36.967 ± 1.384	2.091 ± 0.485***###	90.399 ± 9.127***###
Teprenone	6 h	1.858 ± 0.407***	47.917 ± 1.013***##	3.727 ± 0.441***###	141.38 ± 5.175***###
	24 h	1.388 ± 0.258*	40.75 ± 1.4**#	2.909 ± 0.501***#	93.513 ± 5.762***###
Sodium pyruvate	6 h	1.817 ± 0.422***	49.417 ± 2.219***	3.728 ± 0.442***###	135.851 ± 8.246***###
	24 h	1.49 ± 0.275	37.217 ± 1.412	3.031 ± 0.376***	94.015 ± 6.698***###
N-acetylcysteine	6 h	2.172 ± 0.208**	49.783 ± 1.896***	4.182 ± 0.501***#	139.812 ± 9.524***###
	24 h	1.412 ± 0.394	40.367 ± 1.017**	3.394 ± 0.455***	94.473 ± 8.438***###
Ethyl pyruvate	6 h	2.182 ± 0.781**	48.617 ± 3.244***#	4.03 ± 0.544***##	145.475 ± 6.113***###
	24 h	1.382 ± 0.191*	38.267 ± 1.188*	3.091 ± 0.608***	97.246 ± 4.31***###
Vitamin C	6 h	1.897 ± 0.387***	46.9 ± 2.447***###	3.394 ± 0.647***###	128.464 ± 9.64***###
	24 h	1.395 ± 0.157*	39.633 ± 1.752**	2.636 ± 0.595***##	88.057 ± 6.084***###
Dobutamine	6 h	3.04 ± 0.551	55.917 ± 1.614	5.788 ± 0.519**	178.318 ± 6.016**
	24 h	2.563 ± 0.437	36.1 ± 0.978	4.909 ± 0.575	145.048 ± 6.957***

Compared with the Burn group: ^*^ *P* < 0.05, ^**^ *P* < 0.01, ^***^ *P* < 0.001; Compared with the WHO-ORS group: ^#^ *P* < 0.05, ^##^ *P* < 0.01, ^###^ *P* < 0.001.

Blood lactate levels were significantly elevated in the Burn group at both 6 and 24 h post-injury compared with the Sham group (6 h: 3.277 ± 0.383 vs. 1.118 ± 0.252 mmol/L, *P* < 0.001; 24 h: 2.653 ± 0.534 vs. 1.138 ± 0.196 mmol/L, *P* < 0.05) (Figure 10A). The WHO-ORS group showed a reduction in lactate levels at both time points (6 h: 2.44 ± 0.408 mmol/L; 24 h: 1.922 ± 0.279 mmol/L), but these differences were not statistically significant compared with the burn group. Among the drug-treated groups, glutamine, butyrate, melatonin, teprenone, sodium pyruvate, N-acetylcysteine, ethyl pyruvate, and vitamin C significantly reduced lactate levels at 6 h (*P* < 0.01 for all). At 24 h, these groups continued to show lower lactate levels than did the burn group, with significant differences observed in glutamine, teprenone, ethyl pyruvate, and vitamin C levels (*P* < 0.05). Hypertonic saline and dobutamine did not significantly reduce lactate levels at any time point. Notably, although several drug-treated groups, including glutamine (6 h: 1.845 ± 0.435 mmol/L; 24 h: 1.413 ± 0.226 mmol/L), melatonin (6 h: 1.935 ± 0.51 mmol/L; 24 h: 1.513 ± 0.323 mmol/L), teprenone (6 h: 1.858 ± 0.407 mmol/L; 24 h: 1.388 ± 0.258 mmol/L), sodium pyruvate (6 h: 1.817 ± 0.422 mmol/L; 24 h: 1.49 ± 0.275 mmol/L), and vitamin C (6 h: 1.897 ± 0.387 mmol/L; 24 h: 1.395 ± 0.157 mmol/L), exhibited mean lactate levels within the normal range (<2 mmol/L) and lower than those of the WHO-ORS group, these differences were not statistically significant (*P* > 0.05).

Hematocrit levels significantly increased in the burn group at 6 h post-injury compared with the sham group (58.433% ± 1.788% vs. 39.95% ± 2.669%, *P* < 0.001) (Figure 10B), indicating severe hemoconcentration due to fluid loss and capillary leakage. By 24 h, HCT levels in the burn group decreased to 33.317% ± 1.899%, which was below the normal range (35%–52%), suggesting potential hypovolemia or hemodilution. The WHO-ORS group exhibited elevated HCT levels at 6 h (53.083% ± 1.533%), indicating initial hemoconcentration. By 24 h, HCT levels in the WHO-ORS group decreased to 35.65% ± 2.199%, which was at the lower limit of the normal range, suggesting that fluid resuscitation with WHO-ORS was partially effective but may have approached the threshold of adequacy. Among the drug-treated groups, glutamine, butyrate, melatonin, teprenone, sodium pyruvate, N-acetylcysteine, ethyl pyruvate, and vitamin C significantly reduced the HCT levels at 6 h (*P* < 0.001), with most groups maintaining lower levels at 24 h (*P* < 0.05). Compared to the WHO-ORS group, teprenone (6 h: 47.917% ± 1.013% vs. 53.083% ± 1.533%, *P* < 0.01) and vitamin C (6 h: 46.9% ± 2.447% vs. 53.083% ± 1.533%, *P* < 0.001) showed significantly lower HCT levels at 6 h, indicating their potential efficacy in ameliorating hemoconcentration post-injury. Additionally, butyrate (6 h: 48.683% ± 1.649% vs. 53.083% ± 1.533%, *P* < 0.05), melatonin (6 h: 48.483% ± 1.634% vs. 53.083% ± 1.533%, *P* < 0.05), and ethyl pyruvate (6 h: 48.617% ± 3.244% vs. 53.083% ± 1.533%, *P* < 0.05) also exhibited significantly lower HCT levels at 6 h, though to a lesser extent than did teprenone and vitamin C.

MDA levels, a marker of oxidative stress, were significantly elevated in the Burn group at both time points compared with the Sham group (6 h: 7.091 ± 0.718 vs. 1.697 ± 0.582 nmol/mL, *P* < 0.001; 24 h: 5.667 ± 0.833 vs. 1.758 ± 0.44 nmol/mL, *P* < 0.001) (Figure 10C). The WHO-ORS group showed a reduction in MDA levels at both time points (6 h: 5.455 ± 0.699 nmol/mL, *P* < 0.001 compared with the Burn group; 24 h: 4.152 ± 0.58 nmol/mL, *P* < 0.01 compared with the Burn group). Among the drug-treated groups, glutamine, butyrate, melatonin, teprenone, sodium pyruvate, N-acetylcysteine, ethyl pyruvate, and vitamin C significantly reduced MDA levels at both time points (*P* < 0.001 for all). Compared with the WHO-ORS group, glutamine (6 h: 4.091 ± 0.548 vs. 5.455 ± 0.699 nmol/mL, *P* < 0.01), butyrate (6 h: 4.03 ± 0.48 vs. 5.455 ± 0.699 nmol/mL, *P* < 0.001; 24 h: 3.273 ± 0.754 vs. 4.152 ± 0.58 nmol/mL, *P* < 0.001), melatonin (6 h: 3.637 ± 0.586 vs. 5.455 ± 0.699 nmol/mL, *P* < 0.001; 24 h: 2.091 ± 0.485 vs. 4.152 ± 0.58 nmol/mL, *P* < 0.001), teprenone (6 h: 3.727 ± 0.441 vs. 5.455 ± 0.699 nmol/mL, *P* < 0.001; 24 h: 2.909 ± 0.501 vs. 4.152 ± 0.58 nmol/mL, *P* < 0.05), sodium pyruvate (6 h: 3.728 ± 0.442 vs. 5.455 ± 0.699 nmol/mL, *P* < 0.001), N-acetylcysteine (6 h: 4.182 ± 0.501 vs. 5.455 ± 0.699 nmol/mL, *P* < 0.05), ethyl pyruvate (6 h: 4.03 ± 0.544 vs. 5.455 ± 0.699 nmol/mL, *P* < 0.01), and vitamin C (6 h: 3.394 ± 0.647 vs. 5.455 ± 0.699 nmol/mL, *P* < 0.001; 24 h: 2.636 ± 0.595 vs. 4.152 ± 0.58 nmol/mL, *P* < 0.01) demonstrated significantly lower MDA levels, highlighting their superior antioxidant effects. Hypertonic saline and dobutamine did not significantly reduce MDA levels.

IL-6 levels, a pro-inflammatory cytokine, were significantly elevated in the burn group at both time points compared with the sham group (6 h: 196.923 ± 6.024 vs. 57.994 ± 4.32 pg/mL, *P* < 0.001; 24 h: 175.933 ± 6.699 vs. 56.142 ± 4.543 pg/mL, *P* < 0.001) (Figure 10D). The WHO-ORS group showed a reduction in IL-6 levels at both time points (6 h: 175.761 ± 5.947 pg/mL, *P* < 0.01; 24 h: 147.509 ± 5.952 pg/mL, *P* < 0.001). Hypertonic saline (6 h: 162.433 ± 7.245 pg/mL, *P* < 0.01; 24 h: 133.917 ± 6.123 pg/mL, *P* < 0.01) and dobutamine (6 h: 158.367 ± 6.899 pg/mL, *P* < 0.01; 24 h: 129.783 ± 5.876 pg/mL, *P* < 0.01) also significantly reduced IL-6 levels compared with the burn group, though the reduction at 6 h did not reach the *P* < 0.001 threshold. All other drug-treated groups, including glutamine, butyrate, melatonin, teprenone, sodium pyruvate, N-acetylcysteine, ethyl pyruvate, and vitamin C, showed significantly reduced IL-6 levels at both time points (*P* < 0.001 for all). Compared with the WHO-ORS group, glutamine (6 h: 130.354 ± 11.645 vs. 175.761 ± 5.947 pg/mL, *P* < 0.001; 24 h: 93.92 ± 8.887 vs. 147.509 ± 5.952 pg/mL, *P* < 0.001), butyrate (6 h: 144.649 ± 6.538 vs. 175.761 ± 5.947 pg/mL, *P* < 0.001; 24 h: 99.003 ± 6.33 vs. 147.509 ± 5.952 pg/mL, *P* < 0.001), melatonin (6 h: 130.23 ± 9.201 vs. 175.761 ± 5.947 pg/mL, *P* < 0.001; 24 h: 90.399 ± 9.127 vs. 147.509 ± 5.952 pg/mL, *P* < 0.001), teprenone (6 h: 141.38 ± 5.175 vs. 175.761 ± 5.947 pg/mL, *P* < 0.001; 24 h: 93.513 ± 5.762 vs. 147.509 ± 5.952 pg/mL, *P* < 0.001), sodium pyruvate (6 h: 135.851 ± 8.246 vs. 175.761 ± 5.947 pg/mL, *P* < 0.001; 24 h: 94.015 ± 6.698 vs. 147.509 ± 5.952 pg/mL, *P* < 0.001), N-acetylcysteine (6 h: 139.812 ± 9.524 vs. 175.761 ± 5.947 pg/mL, *P* < 0.001; 24 h: 94.473 ± 8.438 vs. 147.509 ± 5.952 pg/mL, *P* < 0.001), ethyl pyruvate (6 h: 145.475 ± 6.113 vs. 175.761 ± 5.947 pg/mL, *P* < 0.001; 24 h: 97.246 ± 4.31 vs. 147.509 ± 5.952 pg/mL, *P* < 0.001), and vitamin C (6 h: 128.464 ± 9.64 vs. 175.761 ± 5.947 pg/mL, *P* < 0.001; 24 h: 88.057 ± 6.084 vs. 147.509 ± 5.952 pg/mL, *P* < 0.001) exhibited significantly lower IL-6 levels, suggesting their enhanced anti-inflammatory properties.

## 4 Discussion

This study comprehensively investigated the therapeutic potential of various pharmacological interventions as adjuncts to the standard ORT for severe burn injury. By integrating bibliometric analysis, translational validation, and animal experimentation, we identified and validated several promising therapeutic agents.

Given the limited research specifically focusing on ORT for burn shock, the search strategy was expanded to include broader themes related to burn injury, shock, and gastrointestinal protection. By expanding the scope beyond ORT for burn shock, this approach provides a more comprehensive understanding about the broader research landscape. This broader perspective allowed identifying key areas of interest and collaboration within related fields, such as gastrointestinal protection and treatment of typical burn-related symptoms (e.g., ischemia, hypoxia, oxidative stress, and reperfusion injury). This methodological choice not only enriches the analysis but also ensures that the findings are applicable to a wider range of clinical scenarios, thereby enhancing the translational potential of this research.

### 4.1 Integrated bibliometric insights supporting drug prioritization

Our integrated bibliometric analysis systematically mapped the research landscape pertinent to burn injury pathophysiology and therapeutic interventions, specifically targeting mechanisms relevant to ORT enhancement. The fluctuating yet substantial publication output over two decades, with peaks potentially linked to major global health events (Leitmann, 2007; McCall, 2014; Fineberg, 2014; Frieden et al., 2014; Skegg et al., 2021), underscores sustained interest in addressing this critical challenge. Crucially, the geospatial distribution revealed China’s dominant research volume (47.4% of publications) alongside Italy’s exceptional citation impact per paper (83.6 citations/paper), highlighting diverse yet significant contributions to the field’s evidence base. The identification of core dissemination venues such as *Shock*, *Journal of Surgical Research*, and *World Journal of Gastroenterology*, along with pivotal researchers (e.g., Sheng Zhiyong, Cuzzocrea Salvatore, Hu Sen) and their collaborative networks, points to established communities driving knowledge about burn shock and gut protection.

Most critically, keyword analysis confirmed the centrality of our targeted pathophysiological mechanisms: high-frequency terms such as “injury,” “intestinal,” “reperfusion,” and the derived clusters explicitly encompassed ischemia-reperfusion injury, oxidative stress, intestinal barrier dysfunction, fluid resuscitation, and inflammatory pathways. This thematic alignment validates our search strategy for determining the relevant literature spectrum and provides the foundational evidence base from which candidate drugs were systematically identified and prioritized based on research prominence and mechanistic relevance (BES), directly informing our subsequent experimental validation.

This bibliometric approach aligns with established frameworks where co-occurrence networks reflect conceptual knowledge structures (Donthu et al., 2021). The dominance of pathophysiological mechanisms (e.g., ischemia-reperfusion, oxidative stress) parallels trends in nutritional legume research, where keyword clusters similarly highlight core bioactive compounds such as antioxidants and dietary fiber (Akin et al., 2025). Italy’s exceptional citation impact (83.6 citations/paper) despite moderate output further mirrors patterns observed in Canadian pea research, underscoring how targeted collaborations drive disproportionate influence in specialized fields (Akin et al., 2025).

### 4.2 Drug screening and BES evaluation

The BES of drugs based on standardized metrics, including frequency of occurrence, impact scores, publication quality, and journal impact factors, provided a robust framework for identifying the most promising candidates for further investigation. Glutamine emerged as the top candidate, supported by its high frequency of occurrence, substantial impact scores, and a significant number of high-quality publications. This aligns with the existing literature that highlights its role as a critical amino acid in stimulating intestinal immunity and preserving intestinal integrity, particularly in critical care settings (Ren et al., 2016; Achamrah et al., 2017; Cruzat et al., 2018). Hypertonic saline and butyrate also demonstrated potential, albeit to a lesser extent. Hypertonic saline, a hyperosmolar agent, has been shown to improve blood flow distribution and reduce tissue edema, making it particularly relevant in burn injury management (Stefanos et al., 2023). Butyrate, a short-chain fatty acid, plays a key role in ameliorating burn-induced inflammation and intestinal injury, likely through its anti-inflammatory and epithelial protective effects (Luck et al., 2021; Zhou et al., 2021). Melatonin, a potent antioxidant and regulator of circadian rhythms, has shown promise for mitigating oxidative stress and modulating inflammatory responses (Mehrzadi et al., 2023). Teprenone, a gastric mucosal protective agent, is effective in reducing gastric injury and enhancing mucosal defense mechanisms (Zhao et al., 2021; Yao et al., 2024). Sodium pyruvate and ethyl pyruvate, both known for their antioxidant and anti-inflammatory properties, have been identified as potential candidates for reducing oxidative damage and systemic inflammation (Yao et al., 2019). N-acetylcysteine, a precursor of glutathione, exhibits strong antioxidant activity and has the potential to attenuate oxidative stress in burn injuries (Parihar et al., 2008; Raghu et al., 2021). Vitamin C is a well-established antioxidant that scavenges free radicals and supports tissue repair (May and Harrison, 2013). Dobutamine, a beta-adrenergic agonist, has the potential to improve hemodynamic stability (Bruning et al., 2021).

### 4.3 Comprehensive analysis of therapeutic agents in a burn injury rat model

In this study, the effects of the various therapeutic agents mentioned above on the survival rates and biochemical indicators were evaluated in a rat model of burn injury to comprehensively assess their potential therapeutic efficacy and provide critical insights into the pathophysiological responses to burn injury and the therapeutic efficacy of various interventions. Compared with the burn group, the WHO-ORS group demonstrated statistically significant improvements in HCT, MDA, and IL-6 levels at 6 h, as well as in MDA and IL-6 levels at 24 h (*P* < 0.05), indicating that oral rehydration therapy has a measurable therapeutic effect. However, the WHO-ORS failed to achieve significant improvements in 48-h survival, blood lactate levels at 6 and 24 h, and HCT at 24 h compared to the burn group (*P* > 0.05). This suggests that while the WHO-ORS can partially mitigate certain aspects of burn-induced pathophysiology, addressing the complex metabolic dysregulation associated with severe burn injuries is insufficient.

In contrast, pharmacological interventions, such as glutamine, teprenone, ethyl pyruvate, and vitamin C demonstrated statistically significant improvements across all biochemical parameters at both 6 and 24 h compared with the burn group (all, *P* < 0.05). Among these, teprenone and vitamin C stood out as valuable adjuncts to standard rehydration therapy, achieving not only significant improvements in biochemical indicators but also the highest 48-h survival rates (80% and 80%, respectively, *P* < 0.05, compared with the burn group). Teprenone’s exceptional efficacy is particularly notable given its primary clinical application in gastric protection. Experimental evidence suggests its mechanisms may extend to burn shock management through multi-targeted actions: First, it preserves intestinal mucosal barrier integrity by significantly upregulating tight junction proteins including occludin and ZO-1 intestinal barrier integrity by stimulating mucus synthesis and stabilizing tight junctions, which may prevent burn-induced epithelial dissociation and villus necrosis (Guo et al., 2015). Second, it mitigates systemic oxidative stress via upregulation of heat-shock proteins (HSP70/HSP27) that protect enterocytes against hypoxia-reperfusion injury (Kim et al., 2015). Third, it exerts potent anti-inflammatory effects through suppression of NF-κB activation and downstream IL-6/TNF-α cascades (Liu X. et al., 2024), mechanistically explaining the observed reduction in IL-6 versus WHO-ORS (*P* < 0.001). Similarly, vitamin C’s survival benefit is mechanistically linked to its dual antioxidant-anti-inflammatory roles: As a scavenger of reperfusion-generated ROS (Liu et al., 2020), a regenerator of endogenous antioxidants, and a modulator of NF-κB signaling (Xu et al., 2024)—effects that collectively may disrupt the oxidative-inflammatory cascade in burns. These multi-targeted mechanisms directly translate to functional improvements. Teprenone’s barrier stabilization underlies its hematocrit normalization (47.9% vs. 58.4% in Burn at 6 h, *P* < 0.001 and 47.9% vs. 53.1% in WHO-ORS at 6 h, *P* < 0.01). Vitamin C’s ROS scavenging explains superior MDA reduction (3.394 vs. 7.091 nmol/mL in Burn at 6 h, *P* < 0.001 and 3.394 vs. 5.455 nmol/mL in WHO-ORS at 6 h, *P* < 0.001). Both agents’ NF-κB suppression correlates with greater IL-6 reduction (vs. the Burn and WHO-ORS groups; all, *P* < 0.001).

The evaluation of therapeutic agents in this burn injury model revealed critical pathophysiological insights alongside differential treatment efficacy. The untreated burn group exhibited a biphasic hematocrit trajectory—initial hemoconcentration (58.4% at 6 h) progressing to pathological hemodilution (33.3% at 24 h)—reflecting the transition from acute plasma extravasation to systemic inflammatory response syndrome (SIRS)-mediated vasoplegia and circulatory collapse, where effective blood volume depletion occurs despite total body fluid deficit (Zhi et al., 2015; Sen et al., 2019). Against this pathophysiological backdrop, teprenone and vitamin C demonstrated exceptional therapeutic profiles, achieving not only the highest 48-h survival rates (80% each) but also restoring physiological hematocrit levels (40.75% and 39.63% at 24 h, respectively) through microcirculatory stabilization and endothelial protection. Their efficacy stemmed from multi-targeted mechanisms: teprenone restored physiological HCT through microcirculatory stabilization, while vitamin C achieved similar normalization via endothelial protection. In contrast, hypertonic saline exacerbated the pathological hemodilution (34.8% at 24 h), failing to elevate hematocrit above the hypovolemic threshold (<35%) or improve survival (50%, equivalent to WHO-ORS alone), underscoring that mere osmotic fluid shifting cannot reverse the underlying SIRS-mediated vascular dysfunction. This therapeutic dichotomy highlights that successful resuscitation requires addressing the fundamental inflammatory cascade rather than simplistic volume manipulation, with the hematocrit normalization observed in teprenone and vitamin C groups serving as a biomarker of restored microcirculatory integrity rather than isolated hemodilution.

Although glutamine and ethyl pyruvate also demonstrated significant improvements across all biochemical parameters, their 48-h survival rates did not reach statistical significance compared with the burn group (*P* > 0.05). This suggests that although these agents are effective in mitigating specific aspects of burn pathophysiology, their overall therapeutic impact may be less robust than that of teprenone and vitamin C.

Notably, hypertonic saline and dobutamine not only failed to demonstrate significant improvements compared with the burn group in most biochemical parameters, but also underperformed relative to WHO-ORS in certain aspects. For instance, 24 h post-injury, hypertonic saline and dobutamine showed no significant reduction in MDA levels compared with the burn group (*P* > 0.05), whereas WHO-ORS achieved a statistically significant reduction (*P* < 0.01). This further underscores their unsuitability as an adjunct to ORT for burn injury management at the tested doses.

### 4.4 Clinical-translational prioritization framework

To bridge the gap between research activity and clinical deployment, we further developed a three-dimensional evaluation paradigm. The ITS weighted MCAS highest (0.45) to prioritize agents with human clinical evidence, followed by BES (0.35) reflecting research volume and impact, while EDF (0.2) assessed practical deployment. This weighting scheme elevated clinical relevance over bibliometric prominence, with MCAS + EDF (clinical-practical dimensions) collectively constituting 65% of the ITS—ensuring research-active agents without clinical translation potential would be deprioritized. Three key patterns emerged: First, agents with strong research profiles but clinical-practical flaws were selectively demoted: Ethyl pyruvate (BES#8 → ITS#17, Δ = −9) collapsed due to EDF Tier C and moderate MCAS (Z-score = −0.16), predicting its marginal experimental performance (60% survival, *P* > 0.05; biomarker improvements insufficient for statistical significance versus WHO-ORS). Dobutamine (BES#10 → ITS#20, Δ = −10) failed from relatively low MCAS (Z-score = −0.454, weak mechanistic evidence for gut protection) and EDF Tier C, correlating with comprehensive experimental failure (50% survival, no HCT/Lac improvement). Second, high-BES agents with partial clinical deficiencies retained moderate priority but showed limited efficacy: Hypertonic saline (BES#2 → ITS#2) maintained rank due to high MCAS (Z-score = 1.903), but its EDF Tier C foreshadowed suboptimal results: 50% survival (equaling WHO-ORS) and no Lac and MDA reduction (*P* > 0.05 vs. Burn at 24 h), confirming deployability limitations constrain therapeutic impact. Third, the framework correctly validated clinically aligned agents: Vitamin C (BES#9 → ITS#6, Δ = +3) ascended via EDF Tier A and relatively high MCAS (Z-score = −0.09), achieving top-tier efficacy (80% survival *P* < 0.01 vs. Burn). N-acetylcysteine (BES#7 → ITS#3, Δ = +4) leveraged EDF Tier A and relatively high MCAS (Z-score = 0.28) to demonstrate significant anti-inflammatory effects (IL-6 at 24 h *P* < 0.001 vs. Burn). Though teprenone (BES#5 → ITS#9) was penalized by low MCAS (Z-score = −0.60), its EDF Tier A (approved oral drug) enabled selection for testing, where it demonstrated 80% survival and comprehensive biomarker normalization.

### 4.5 Limitations

Despite these promising results, this study has some limitations that warrant consideration. First, the BES evaluation, although methodologically robust, is inherently constrained by the quality and availability of the underlying data. Reliance on metrics, such as frequency of occurrence, impact scores, and journal impact factors may introduce bias, as these parameters do not fully capture the clinical relevance, mechanistic depth, or translational potential of the reviewed studies. Second, the animal experiments, though informative, have inherent limitations regarding translatability to human patients. The rat models used in this study, although widely accepted for burn injury research, may not fully replicate the complex pathophysiology of human burn injuries, particularly in terms of systemic inflammatory responses and long-term metabolic dysregulation. Additionally, the short duration of the experiments (48 h) may not have captured long-term therapeutic effects or potential adverse reactions of the drugs. Third, this study focused on a limited set of biomarkers (Lac, HCT, MDA, and IL-6) to assess drug efficacy. Although these biomarkers are well-established indicators of tissue oxygenation, oxidative stress, and inflammation, they do not provide a comprehensive picture of the drugs’ mechanisms of action or potential side effects. Future studies should incorporate a broader range of biomarkers, such as mitochondrial function markers, endothelial integrity markers, and advanced inflammatory cytokines to fully elucidate the therapeutic potential of these drugs. Additionally, integrating histological examinations such as tissue pathology analysis could provide critical insights into the structural and cellular changes induced by burn injury and the therapeutic effects of drugs. Fourth, this study used a single dose of each drug based on the most commonly used dose in rat models. While this approach allows for rapid screening of the most effective agents, it does not account for potential dose-dependent effects or identify the optimal therapeutic dose for each drug in the context of severe burn injury. Future research should include dose-response studies to determine the most effective and safe dosage regimens for these agents, particularly in the context of oral rehydration therapy for managing patients with burns. Fifth, although the ITS weighting (MCAS 0.45/BES 0.35/EDF 0.2) reflected our emphasis on clinical evidence, alternative weightings could be explored—particularly increasing EDF for austere environments. Future implementations may adjust weights based on specific operational contexts. Sixth, animal experiments prioritized BES Top 10 agents, excluding high-ITS candidates like Pentoxifylline (ITS#6) and Dexmedetomidine (ITS#10) that entered ITS Top 10, while agents demoted from BES Top 10 (e.g., ethyl pyruvate ITS#17, dobutamine ITS#20) were still tested due to initial BES selection. Future studies should directly evaluate high-ITS agents to fully validate the framework. Lastly, the study did not explore potential drug-drug interactions or the effects of combination therapy. Given the complexity of critical care settings, in which patients often receive multiple medications, future research should investigate the synergistic or antagonistic effects of combining these drugs with other commonly used therapies.

## 5 Conclusion

This study highlights the limitations of the WHO-ORS as a stand-alone therapy for severe burn injuries and underscores the potential of pharmacological adjuncts, particularly teprenone and vitamin C, to enhance therapeutic outcomes. Their demonstrated efficacy in improving survival rates and mitigating secondary complications provides a rationale for their clinical applications. However, further studies are needed to elucidate their precise mechanisms of action, optimize dosing regimens, and evaluate their clinical applicability in patients with burns.

## Data Availability

The original contributions presented in the study are included in the article/supplementary material, further inquiries can be directed to the corresponding authors.
