# Correction: Bibliometric analysis and initial animal efficacy evaluation of top ten scoring drugs to enhance oral rehydration therapy in early post-burn shock

**DOI:** 10.3389/fphar.2025.1750337

**Published:** 2025-12-11

**Authors:** Xiang-Yu Liu, Yu-Shou Wu, Yi-Rui Qu, Hui Zhou, Tian Liu, Xiao-Wei Su, Fang-Chao Hu, Jin-Guang Zheng, Shao-Fang Han, Jia-Ke Chai, Yun-Fei Chi

**Affiliations:** 1 Senior Department of Burns & Plastic Surgery, Institute of Burn in the Fourth Medical Centre, Chinese PLA General Hospital, Beijing, China; 2 Graduate School, Chinese PLA General Hospital, Beijing, China

**Keywords:** burn, shock, oral rehydration therapy, world health organization-recommended oral rehydration solution, drug screening, bibliometric analysis, animal experiment, clinical application

The order of Affiliation 1 and 2 was published incorrectly. “Senior Department of Burns & Plastic Surgery, Institute of Burn in the Fourth Medical Centre, Chinese PLA General Hospital, Beijing, China” is updated as **Affiliation 1**, while “Graduate School, Chinese PLA General Hospital, Beijing, China” is updated as **Affiliation 2**.

The original article has been updated.

